# Development and Psychometric Validation of the Patient Experience for Disease Management Scale (PEDMS) in Outpatient Settings in Najran, Saudi Arabia

**DOI:** 10.3390/healthcare13101191

**Published:** 2025-05-20

**Authors:** Nasser Saeed Alqahtani

**Affiliations:** Department of Family and Community Medicine, College of Medicine, Najran University, Najran 66462, Saudi Arabia; drnasser1000@hotmail.com

**Keywords:** psychometric validation, patient experience, disease management, psychometric evaluation, scale development, healthcare, outpatient settings

## Abstract

**Background**: Measuring patient experience is essential for enhancing healthcare quality, particularly in disease management contexts. Existing instruments often lack specificity for outpatient settings. **Objective**: The objective of this study is to create and assess the Patient Experience for Disease Management Scale (PEDMS) psychometrically in order to precisely record patient experiences. **Method**: A mixed-methods approach was used, with qualitative insights derived from the literature and expert consultations, followed by quantitative analyses. A sample of 600 outpatients was surveyed in Najran, Saudi Arabia, from September 2023 to May 2024, using exploratory and confirmatory factor analyses (CFA) to test the scale’s psychometric qualities. **Results**: The PEDMS identified eight distinct factors, explaining 86.32% of the variance. The findings from the CFA significantly affirm the validity of the PEDMS’s structure, demonstrating excellent model fit as indicated by multiple fit indices: CFI (0.965), NFI (0.975), SRMR (0.051), and RMSEA (0.067). Additionally, the PEDMS exhibits strong convergent validity, supported by high CR and AVE values, both exceeding 0.70 and 0.50, respectively. Furthermore, all item loadings are greater than 0.70, reinforcing the PEDMS’s robustness. The reliability of the PEDMS is considered excellent, as demonstrated by a Cronbach’s alpha coefficient of 0.878 for the entire 38-item scale. Each factor exhibits strong internal consistency, with values ranging from 0.827 to 0.879, all surpassing the threshold of 0.7. **Conclusions**: The PEDMS provides a reliable tool for measuring patient experiences in disease management, offering healthcare providers actionable insights for patient-centered care. This study emphasizes the need to integrate standardized metrics into quality improvement frameworks in order to improve healthcare outcomes.

## 1. Introduction

Measuring patient experience is increasingly recognized as essential to healthcare quality, particularly in the context of disease management. Patient experience encompasses various factors, including communication with providers, accessibility of care, and overall satisfaction with treatment [1]. Research demonstrates a strong correlation between positive patient experiences and improved clinical outcomes, highlighting the necessity of understanding patient perspectives [2]. Patient experience serves as a key quality indicator, with evidence linking it to patient safety and clinical effectiveness across various conditions [3]. Positive experiences contribute to better adherence to treatment plans, while negative experiences can increase the risk of nonadherence by 19% [4,5]. Furthermore, enhancing patient experiences can lead to reduced hospital readmissions and lower healthcare costs [6]. Qualitative research indicates that treatment burdens and psychological challenges are often exacerbated by poor communication and a lack of patient involvement in their care [7].

This growing body of evidence underscores the importance of integrating patient experience metrics into quality improvement frameworks to enhance health outcomes and healthcare efficiency. Current discussions focus on the most effective methods for measuring patient experience and its impact on clinical outcomes. Some studies advocate for comprehensive approaches, while others suggest that streamlined measures can yield positive results [8,9,10,11]. By utilizing standardized measures, healthcare providers can gain actionable insights to inform quality improvement in health services.

However, significant gaps persist in the development and validation of relevant instruments specifically for outpatients. While more studies are needed to refine the measurement of patient experience [12], it is essential to acknowledge that there is no “one-size-fits-all” solution. A diverse array of instruments exists, each with its own strengths and limitations in utility [13]. A systematic review underscores the necessity for tools that concentrate on clearly defined concepts and dimensions, emphasizing the importance of precision in measuring patient experiences [14]. Many patient experience assessment tools are primarily intended for inpatient settings, where patient care, interactions, and healthcare processes differ dramatically from those in outpatient settings. Outpatient care does not often include longer stays, more intensive treatments, or constant monitoring, but inpatient care does. Outpatient settings provide a diverse range of services, from routine check-ups to specialty therapies, making it difficult to create a one-size-fits-all tool. Outpatient care’s diversity necessitates the use of instruments capable of capturing individual complexities and variances in patient experiences across various types of services [12]. Furthermore, outpatient settings have specific issues such as appointment scheduling, waiting periods, and health care coordination across various providers. These elements are critical to the patient experience, but they may be neglected by tools that do not have a holistic approach. By developing the Patient Experience for Disease Management Scale (PEDMS), we aim to address these gaps and provide a tool that accurately reflects the specific experiences of patients in outpatient settings.

In Saudi Arabia, the necessity of assessing patient experience is becoming more widely recognized as a vital component of healthcare quality, particularly in the context of disease management. Positive patient experiences are substantially linked to improved clinical outcomes, better treatment adherence, and fewer hospital readmissions [1,2].

Saudi Arabia’s Vision 2030 outlines a comprehensive plan to transform the healthcare sector, emphasizing innovation, financial sustainability, and disease prevention [3]. The Health Sector Transformation Program under Vision 2030 aims to create a more effective, integrated, and patient-centered healthcare system. By focusing on patient experience, the PEDMS aligns with Vision 2030’s goals of improving the quality of care and ensuring that healthcare services are responsive to patient needs and preferences.

Nowadays, the healthcare system faces many challenges in meeting end-user satisfaction and improving health outcomes. The PEDMS is designed to address these specific needs by providing a standardized measure of patient experience that can be used to identify areas for improvement. This localized approach ensures that the scale is relevant and applicable to the specific context of Saudi Arabia, ultimately contributing to better healthcare outcomes for its residents.

This study aims to address existing gaps in measuring patient experiences in outpatient settings by developing and psychometrically validating the Patient Experience for Disease Management Scale (PEDMS). The PEDMS is designed to provide healthcare providers with a reliable and valid tool for accurately capturing patient experiences, which can inform quality improvement initiatives and enhance patient-centered care. By employing standardized approaches in scale development and rigorous psychometric evaluation, this research seeks to ensure the reliability and validity of patient experience data. Ultimately, the implementation of PEDMS aims to improve healthcare quality and patient outcomes by providing valuable insights that can guide disease management strategies and patient-centered care in outpatient clinical settings.

To ensure the PEDMS accurately captures patient experiences in outpatient settings, this study employed a rigorous validation process that included several key methods. Exploratory Factor Analysis (EFA) was conducted to identify the underlying factor structure of the scale and determine the number of factors to retain. Confirmatory Factor Analysis (CFA) was used to test the hypothesized factor structure identified by the EFA and assess the model fit using various fit indices. The extraction of factors and item reduction analysis involved refining the scale by removing items that did not contribute significantly to the overall construct, ensuring a concise and focused instrument. The reliability of the scale was evaluated using Cronbach’s alpha to measure internal consistency, ensuring that the items within each factor consistently measured the same construct. Additionally, the validity of the PEDMS was assessed through convergent and discriminant validity tests, ensuring that the scale accurately measures the intended constructs and distinguishes between different constructs. By incorporating these methods, we aimed to develop a robust and reliable tool for measuring patient experiences in disease management within outpatient settings.

## 2. Methodology

### 2.1. Study Design, Setting, and Timing

To develop and assess the psychometric features of PEDMS, this study employed a combined qualitative and quantitative approach. This process was conducted in multiple phases through a series of consecutive steps at Najran University Hospital. The study period spanned from September 2023 to May 2024, aiming to ensure rational scale development and effectively measure the psychometric properties of the scale.

### 2.2. Scale Development

**Figure 1:**
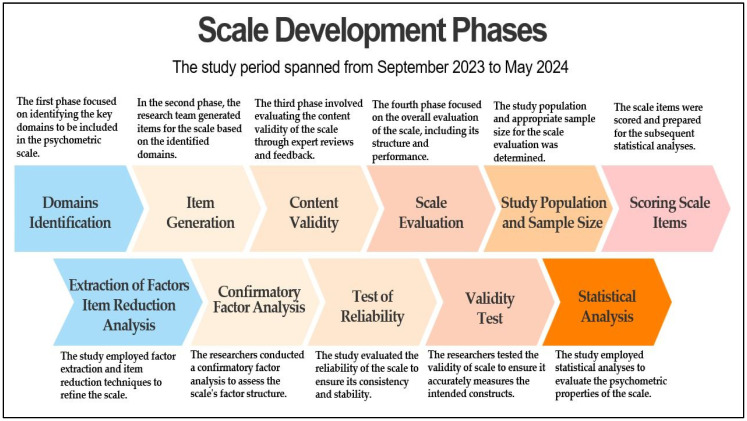
Flowchart depicting various scale development phases.

The PEDMS has been developed through a multi-phase process, as illustrated in Figure 1.

### 2.3. Domain Identification

An extensive review of the existing literature was conducted to identify key dimensions of patient experience relevant to disease management. This study utilized fundamental principles of patient management, along with a framework of various management tasks as described in Robin Fraser’s book “Clinical Method”, represented by the acronym RAPRIOP [15,16,17].

### 2.4. Item Generation

This process was guided by the literature review and the framework established during the domain identification phase. The process of item generation was guided by established best practices in psychometric research. According to DeVellis (2016), effective item generation involves defining the construct, generating a comprehensive item pool, and ensuring that items are clear and relevant [18]. Additionally, Boateng et al. (2018) emphasize the importance of expert consultations and pilot testing to refine items [19]. For each identified domain, specific items were formulated to capture its essence, ensuring relevance and representation of the defined concepts. To enhance clarity, consultations were held with three experts in family medicine. The experts in family medicine were recruited through a targeted selection process at Najran University Hospital. These experts were chosen based on their extensive experience and expertise in family medicine, as well as their active involvement in patient care and research. The recruitment process involved identifying professionals who had a strong background in outpatient care and disease management. Invitations were extended to these experts, and their participation was confirmed through formal agreements. Their feedback refined the wording and focus of the items to accurately reflect patients’ experiences.

In-depth focus groups with final-year medical students and general practitioners gathered insights into their perceptions of the domains and solicited suggestions for optimal item formulation. The final-year medical students and general practitioners were recruited through a targeted selection process at Najran University Hospital. The medical students were selected from the final-year cohort at Najran University, ensuring they had sufficient clinical experience and knowledge to provide valuable insights. Additionally, face-to-face interviews with 15 patients from the target population (who were not included in the final study) explored their understanding and provided feedback, informing necessary adjustments to the draft. The final set of items was developed based on expert feedback and qualitative data analysis. Each item was crafted to ensure clarity, conciseness, and a direct connection to the corresponding domain. Respondent burden varies according to survey length; hence, each item was kept as short as possible to save time for patients (or proxies if patients are unable to complete it themselves), ensuring brevity. Word count is a basic measure of brevity, and only simple words were used, such that the target group understood them all the same.

### 2.5. Content Validity

To establish and enhance the content validity of the PEDMS, the following steps were taken:

**Expert Review**: A panel of five independent experts in patient management and psychometrics was assembled to evaluate the clarity, relevance, comprehensiveness, applicability, and understanding of each item in relation to the identified domains. The panel of experts in patient management and psychometrics was recruited through a targeted selection process at Najran University Hospital. Their evaluations were crucial in determining whether the items adequately represented the constructs they were intended to measure. To minimize bias in the assessment, expert judgment was conducted independently and systematically. The experts’ assessments were quantified using a formalized scaling system as follows: **0**: Not fit at all, **1**: Fits but needs major modifications, **2**: Fits with minor modifications, **3**: Fits, and **4**: Suggested item.

Content validity index at item level (I-CVI): The content validity index is the most frequently used method for content validity in instrument development reports. The expert panel members were requested to score instrument items based on their clarity and relevance to the construct underlying the investigation, as well as the construct’s theoretical definitions and dimensions, using the previously indicated ordinal scale. To calculate the content validity index for relevancy and clarity of each item (I-CVIs), the number of those who rated the item as relevant or clear (rating 3 or 4) was divided by the number of content experts. I-CVI is calculated at the item level by dividing the number of experts who rate the relevancy of each item as 3 or 4 by the total number of experts (*n* = 5 in our study). The I-CVI expresses the proportion of agreement on the relevancy of each item, which is between zero and one. In our study, the I-CVI threshold was ≥0.6 for acceptable agreement. Any item with a lower value (<6) was discarded.

**Evaluation by Target Population**: Following the expert review, the same group of patients engaged in the previous step were invited to evaluate the face validity of the PEDMS. They assessed whether the scale items were appropriate for the targeted construct and aligned with the assessment objectives. Additionally, cognitive interviews were conducted using a think-aloud protocol, allowing the research team to gain insights into how respondents interpreted the items and the reasoning behind their responses. Based on this feedback, necessary modifications were made, particularly to the following four items: specifically, item one and item three in the domain of reassurance/explanation, item four in the domain of advice/counseling, and item four in the domain of prevention/promotion.

Pre Test: A pilot study involving 30 patients, who were excluded from the main study sample, was conducted to evaluate the clarity, appropriateness, acceptability, and response time for each item of the draft PEDMS. Participants were recruited from the outpatients attending Najran University Hospital to represent a diverse range of health conditions and demographic characteristics, including age, gender, and socioeconomic status.

Finalization of the Scale: Based on the steps outlined above, the PEDMS was refined to enhance its validity and reliability for future use in assessing disease management from the patient’s perspective.

### 2.6. Scale Evaluation

#### 2.6.1. Determining the Type of Data and Data Collection Design

This study established that quantitative data are essential for accurately measuring the constructs of interest—specifically, patient experience in disease management—and for assessing the validity and reliability of the scale. A descriptive analytical cross-sectional design was employed for data collection, utilizing interviews administered by well-trained nurses who are familiar with the scale. The term “well-trained nurse” refers to nurses who have received specific training on administering the Patient Experience for Disease Management Scale (PEDMS) and are familiar with its content and purpose. These nurses were recruited from Najran University Hospital, where they were selected based on their experience in outpatient care and their ability to effectively communicate with patients.

#### 2.6.2. Establishing the Study Population and Sample Size

The sample involved a diverse group of outpatients attending Najran University Hospital, considering demographic factors such as age, gender, education, and more. Simple random sampling collected data from fifteen different clinics, including the emergency department, which represented a comprehensive range of health services across various medical specialties during the study period. This diverse setting enhances the generalizability of the findings by reflecting a wide array of medical conditions and treatment experiences.

To minimize measurement errors and achieve more stable factor loadings, a sample size of 600 was selected. This sample was evenly divided, with half allocated for exploratory factor analysis (EFA) and the other half for confirmatory factor analysis (CFA). According to the literature, a sample size of at least 300 is recommended for exploratory factor analysis (EFA) to ensure reliable factor extraction and stable loadings [20]. For confirmatory factor analysis (CFA), larger sample sizes are often required to achieve adequate power and precise parameter estimates [21]. Studies have shown that sample sizes of 500 or more are ideal for complex models with multiple factors [22]. Additionally, a review of psychometric validation studies indicates that sample sizes ranging from 300 to 600 are commonly used to ensure robust and reliable results [20].

#### 2.6.3. Scoring Scale Items

Each item in the PEDMS was carefully crafted to assess specific aspects of disease management, capturing patient experiences related to their care. A Likert-type scale was used for scoring, allowing respondents to indicate their level of agreement, with response options ranging from one (strongly disagree) to five (strongly agree). The scoring for each item was as follows: Strongly disagree: 1 point. Disagree: 2 points. Neutral: 3 points. Agree: 4 points. Strongly agree: 5 points. The total score for each construct was calculated by summing the scores of the relevant items, with higher total scores indicating greater levels of positive experiences in disease management.

### 2.7. Extraction of Factors and Item Reduction Analysis

The extraction of factors helps identify the underlying constructs measured by the scale using exploratory factor analysis (EFA). Before conducting the analysis, the collected data were screened for normality, linearity, and homoscedasticity. The validity of the factor analysis was based on the Kaiser–Meyer–Olkin (KMO) Measure of Sampling Adequacy and Bartlett’s Test of Sphericity (BTOS). Acceptance criteria were a KMO value above 0.60 and a *p*-value less than 0.05 for the BTOS test [23]. The number of factors to retain was determined using the eigenvalue-greater-than-one rule and scree plot analysis. An oblique rotation method was applied to enhance interpretability. Factor loadings of 0.50 or higher were considered significant indicators, while items with lower loadings or cross-loadings were reviewed for potential removal.

### 2.8. Confirmatory Factor Analysis (CFA)

The stability and reliability of the hypothesized factor structure identified in the exploratory phase were further assessed using confirmatory factor analysis (CFA) to test the dimensionality of the scale on a new sample. Maximum likelihood estimation (MLE) was used to estimate the model parameters. The model fit was assessed using several fit indices to determine how well the specified model represented the data. The following indices (specified by Hu and Bentler, 1999) [24] were considered: comparative fit index (CFI): values above 0.90 indicate a good fit. Root mean square error of approximation (RMSEA): values below 0.06 suggest a close fit, and values below 0.08 indicate acceptable fit. Standardized root mean square residual (SRMR): values below 0.08 are considered indicative of a good fit. If the initial model did not fit well, potential improvements were identified to enhance the model’s adequacy.

### 2.9. Test of Reliability

To ensure that the PEDMS serves as a reliable tool for measuring constructs related to patient experiences, this study evaluated its reliability through internal consistency. Cronbach’s alpha was calculated for each factor identified in the exploratory factor analysis. A Cronbach’s alpha value of 0.70 or higher was deemed acceptable, indicating that the items within each factor consistently measure the same underlying construct. Nunnally (1978) suggested that instruments used in basic research should have a reliability of about 0.7 or higher [25].

### 2.10. Validity Test

Validity is an ongoing process that begins with identifying and defining the scale’s domain through content and face validity (see above; scale development, step 3). Further evaluation of construct validity was conducted using EFA and CFA. EFA provided insights into construct validity; if the identified factors aligned with theoretical expectations, this supported the scale’s validity. Conversely, unexpected factors or low loadings indicated potential issues with the items or scale design. Following EFA, CFA was performed to confirm that the scale accurately measures the intended constructs. A well-fitting CFA model, characterized by strong factor loadings and favorable fit indices, provides robust evidence for construct validity. Additionally, to evaluate convergent validity, the following three criteria from Hair et al. (2014) [26] were applied: (1) Item Loadings: Ideally, all item loadings should exceed 0.70, indicating strong alignment with the construct. (2) An AVE of 0.50 or higher is necessary, showing that the items explain more than 50% of the variance, exceeding measurement error. (3) A CR of 0.70 or above is preferred, with values between 0.60 and 0.70 considered acceptable. Meeting these criteria confirms that the items effectively measure the same construct, thereby supporting the scale’s validity.

### 2.11. Statistical Analysis

Statistical analysis for the PEDMS was conducted using IBM SPSS Statistics for Windows (Version 29.0) (IBM Corp., Armonk, NY, USA) and AMOS (Version 26.0).

Descriptive Statistics: Summarized demographic characteristics and the distribution of scale items (means, standard deviations, frequency distributions).

Exploratory Factor Analysis (EFA) was performed, which is a statistical technique commonly used in the social sciences to identify the underlying factor structure of the scale. Using principal component analysis with Varimax rotation. Criteria included an eigenvalue > 1, factor loading > 0.50, and no cross-loading > 0.50 [27,28].

Confirmatory Factor Analysis (CFA): Conducted with AMOS to test the hypothesized factor structure, assess model fit, and construct validity. Key fit indices included CMIN/DF (1–3), CFI (>0.90 for acceptable and >0.95 for excellent), and RMSEA (<0.08 for good fit).

## 3. Results

### 3.1. Characteristics of Study Sample

The results depicted in Table 1 summarize the characteristics of the study sample of 600 individuals. The results showed that the majority of the sample was female (59.5%), with an average age of 33.74 ± 14.69 years, with a large concentration in the age group between 21 and 40 years (46.8%). The results also indicated that the vast majority of participants were married (64.3%) and living in urban areas (40.8%) and that most families were extended (85.3%) and consisted of 3 to 7 individuals (74.2%). In addition, the results showed that the majority of the sample were Saudis (56%) with a university education (48.7%) and that the majority of them were unemployed (52.3%) and economically middle class (56.3%) (see Table 1).

### 3.2. Domain Identification

The identified domains included reassurance/explanation, advice/counseling, prescribing, red flags/referral, investigation, observation/follow-up, prevention/promotion, and patient wants/needs/satisfaction. Once the domains were established, conceptual descriptions for each domain were specified (Table 2).

### 3.3. Pre-Test Results (Pilot Study)

The pilot study results showed that participants had no difficulties understanding the items or identifying awkward terminology, except for three items, which were revised for improved clarity. Additionally, the average time required to complete the scale was approximately 5 to 10 min, indicating that the scale is both clear and reliable. This effectively captures the breadth and depth of patients’ experiences related to disease management. The finalized scale consists of 38 items distributed across eight sections: Reassurance and Explanation (eight items), Advice and Counseling (five items), Prescribing (five items), Red Flags and Referral (five items), Investigation (four items), Observation and Follow-up (three items), Prevention and Promotion (five items), and Patient’s Wants, Needs, and Satisfaction (three items). In addition, to ensure consistency and alignment between the Arabic and English versions, the scale underwent a translation process using the translate-back-translate technique.

### 3.4. Content Validity Index at Item Level (I-CVI) Score

To obtain a content validity index for each item, the number of expert panel members judging the item as relevant was divided by the number of content experts (*n* = 5). In the case of the I-CVI score, all 38 items across eight sections were found to be above the acceptable cut-off level of 0.6. The majority of the items had an I-CVI score of 0.8, which indicates that four out of the five expert panel members rated the relevancy of each item as 3 or 4.

### 3.5. Exploratory Factor Analysis (EFA)

Half of the sample size, estimated at 300 participants, was used for the EFA, while the other half was utilized for the CFA. The EFA identified eight factors, each with Eigen values greater than one, explaining a total of 86.32% of the variance. The Kaiser–Meyer–Olkin (KMO) measure was 0.958, indicating an excellent level of sampling adequacy according to Kaiser (1974) [29]. The Measure of Sampling Adequacy (MSA) was above 0.7 for all 38 items, indicating good sampling adequacy and suitability for factor analysis as mentioned by Hair et al. [26]. Additionally, Bartlett’s Test of Sphericity was significant (χ^2^ = 16,308.474, *p* = 0.00), confirming the appropriateness of the data for factor analysis. Based on the rotated component matrix, Table 3 reveals that the EFA procedure successfully grouped the eight factors into the 38 items. These results provide valuable insights into the interrelationships among different aspects of patient experience, highlighting key areas for improvement in the developed scale. Generally, a factor loading above 0.60 is considered significant, and the high loadings observed for several items suggest a strong association with their respective factors. Notably, all items in the developed scale exhibit high factor loadings with no cross-loadings, confirming their relevance and suitability for retention. These findings are significant indicators of the PEDMS’s efficiency, thereby maintaining its validity and reliability. Overall, the EFA results demonstrate a well-structured scale that effectively captures various dimensions of patient experience related to management plans.

### 3.6. Confirmatory Factor Analysis (CFA)

The findings from the second part of the analyzed data (*n* = 300) are based on the CFA approach. CFA tests the hypothesized factor structure identified in the exploratory phase by examining the overall model fit, as well as the construct validity and reliability of the measured model. The CFA results indicate that the developed scale demonstrates excellent model fit. Various fit indices, including CFI (0.965), NFI (0.975), SRMR (0.051), and RMSEA (0.067), collectively suggest that the model is appropriate for the data (see Table 4 for more details).

**Figure 2:**
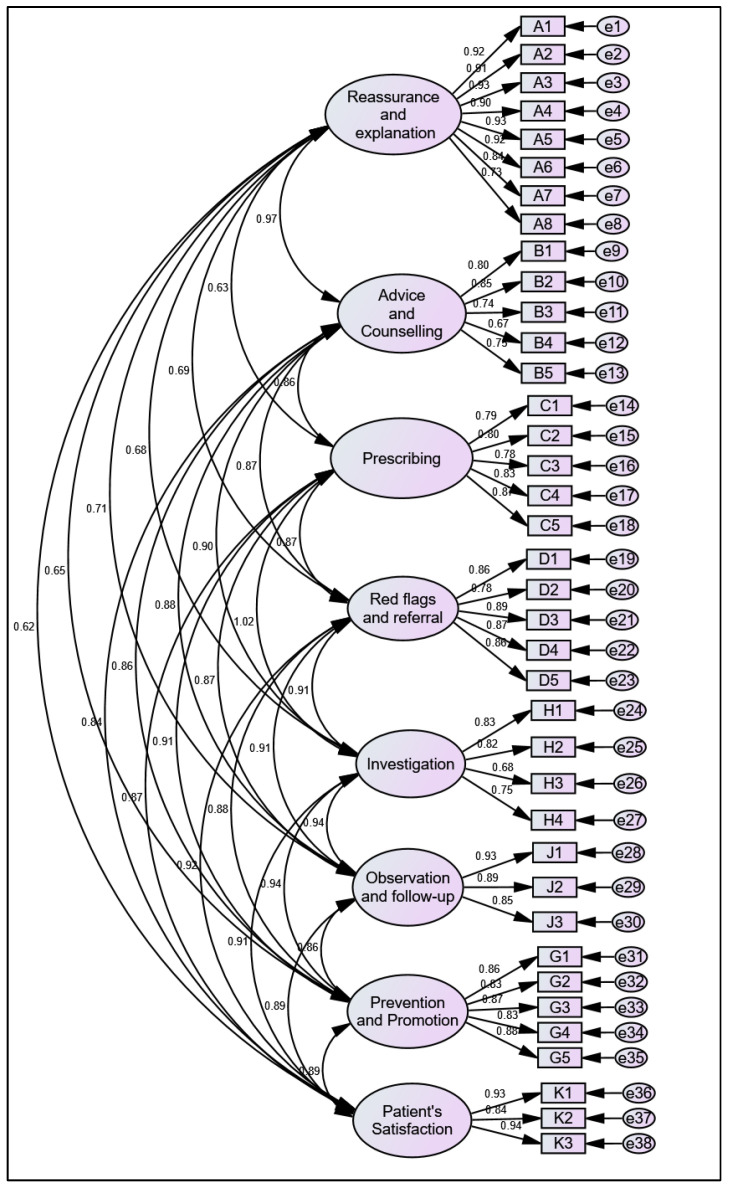
Confirmatory factor analysis model.

After confirming the results of the 8-factor model, convergent validity was assessed using the cutoff points detailed by Hair et al., 2014 [26]. Overall, the criteria for convergent validity in the CFA alternate measurement model were satisfied. The item loadings displayed in the CFA measurement model (Figure 2), along with the AVE and CR, met the preferred values (see Table 5 for more details). Thus, fulfilling these criteria confirms that the items effectively measure the same construct, thereby supporting the PEDMS’s validity.

### 3.7. Reliability Test

The reliability calculation revealed a Cronbach’s alpha coefficient of 0.878 for the entire scale (38 items), which exceeds the acceptable threshold of 0.70. The alpha values ranged from 0.827 to 0.879, indicating sufficient reliability. Furthermore, the “Cronbach’s Alpha if Item Deleted” values suggest that removing any specific item would not significantly enhance overall reliability, highlighting the importance of each item in the PEDMS (see Table 3 for more details).

## 4. Discussion

In the evolving landscape of healthcare, understanding and enhancing patient experiences is paramount, particularly within the realm of disease management. The development and validation of the Patient Experience for Disease Management Scale (PEDMS) serves as a significant advancement in this domain, addressing a critical gap in existing measurement tools that often lack specificity for outpatient settings. This study details the development and evaluation of a robust PEDMS specifically designed to measure patient experience in disease management. Employing a combined qualitative and steps approach facilitates a comprehensive understanding of the issues while amplifying the voices of participants [30,31]. By targeting outpatients with diverse demographic characteristics across various medical settings and disciplines, the PEDMS serves as a vital tool for assessing the quality of care delivered. The insights obtained from the PEDMS are essential for understanding patient perspectives and enhancing healthcare practices. Furthermore, as the integration of management plan guidance is increasingly recognized as a fundamental aspect of effective disease management, the significance of the PEDMS in daily clinical practice is paramount.

The findings of EFA revealed eight distinct factors that collectively accounted for 86.32% of the total variance, demonstrating excellent sampling adequacy and suitability for factor analysis, as confirmed by standard parameters. The MSA value was above 0.7 for all 38 items, and BTOS yielded χ^2^ = 16,308.474, *p* = 0.00, indicating significance. This analysis validated the relevance and suitability of the items for retention, as all factors had eigenvalues greater than 1, and each item exhibited high factor loadings without any cross-loadings detected. These results indicate that the PEDMS is a well-structured instrument comprising 38 items allocated into eight factors, each named according to its content as follows: “Reassurance and Explanation”, “Advice and Counseling”, “Prescribing”, “Red Flags and Referral”, “Investigation”, “Observation and Follow-up”, “Prevention and Promotion”, and “Patient’s Wants, Needs, and Satisfaction”. Each factor effectively captures various dimensions of patient experience related to management plans.

Factor 1, titled “Reassurance and Explanation”, encompasses the following eight key elements: 1. The doctor treats my need for reassurance as the primary reason for seeking medical services. 2. I receive appropriate reassurance that maintains the doctor’s credibility. 3. The doctor provides effective reassurance through thorough history taking and examination. 4. The doctor offers an appropriate degree of explanation regarding my illness. 5. The doctor explores my understanding and fears concerning the symptoms. 6. I trust the doctor due to effective communication. 7. The doctor considers my understanding, education, cultural background, medical experience, and personality. 8. I have a strong bond with the doctor based on continuity of care. This factor underscores the importance of providing patients with clear and concise information about their health conditions and treatment plans, along with realistic hope and emotional support. Such an approach empowers patients to actively participate in the success of their treatment plans. Informed patients are more likely to take responsibility for their health and engage in shared decision-making [32].

Factor 2, titled “Advice and Counseling”, includes five critical elements: 1. The doctor’s advice is realistically adapted to my circumstances, lifestyle, and personality. 2. The doctor helps me identify the physical, psychological, and social aspects of my illness. 3. The doctor assists me in implementing my own solutions for my condition by providing insights and identifying possible actions. 4. The doctor convinces me that I am not physically or psychologically ill; rather, I am facing challenges in adapting to or coping with everyday problems. 5. The doctor counsels me to recognize the need to modify unhealthy behaviors. This factor emphasizes the significance of tailoring recommendations based on individual patient needs, preferences, and circumstances, enhancing overall well-being and empowering patients to take an active role in their care. Research indicates that effective patient education enhances satisfaction and reassurance [33,34]. Furthermore, it is estimated that approximately $100 billion could be saved annually by improving compliance through better advising and education [35].

Factor 3, titled “Prescribing”, comprises five items: 1. I believe the doctor considers any warnings regarding medication safety before prescribing. 2. I think the doctor prescribes the most effective medication. 3. I believe the doctor takes into account the availability and affordability of medication before prescribing. 4. The doctor informs me about the possible adverse effects of medication prior to prescribing. 5. The doctor instructs me on the dosage, timing, and route of administration for the medication. This factor highlights the importance of selecting appropriate medications, determining correct dosages, providing clear instructions for use, discussing potential side effects, and emphasizing the significance of adherence. Safe and effective medication prescribing is essential for achieving optimal health outcomes [36].

Factor 4, titled “Red Flags and Referral”, consists of five items: 1. The doctor makes me aware of the signs that may indicate serious progression or complications that could occur. 2. The doctor explains how I can manage if I encounter any potential serious progression or complications. 3. I believe the doctor arranges appropriate referrals when necessary. 4. The doctor details the referral process, including why, to whom, when, and where the referral will take place. 5. The doctor seeks feedback regarding the referral. This factor emphasizes the critical importance of a proactive approach that empowers patients to recognize warning signs indicating potentially serious underlying conditions requiring further evaluation. It also ensures that patients understand the rationale for referrals, which can lead to early treatment and limit complications. For better health outcomes, it is essential for both at-risk patients and medical practitioners to be aware of potential early warning signs [37].

Factor 5, titled “Investigation”, includes four items: 1. I believe the doctor requests appropriate investigations. 2. I think the doctor considers the risks and costs justified by the value of the information likely to be gained from the tests. 3. I understand the purpose of the requested investigations. 4. The doctor discusses with me what is being looked for in the results. This factor ensures that patients comprehend the purpose and significance of the tests, imaging, and procedures being conducted. Patients who underwent investigations reported lower levels of anxiety when they had a clear understanding of the procedure [38].

Factor 6, titled “Observation and Follow-up”, encompasses three key elements: 1. The doctor encourages follow-up visits to monitor progress. 2. I believe the doctor schedules appropriate appointments for follow-up. 3. The doctor effectively addresses any issues related to follow-up. This factor highlights the importance of the ongoing process of monitoring a patient’s condition, which involves detecting any changes in health status and making necessary adjustments to the treatment plan. Recognizing that health is a lifelong concern, planned follow-up is essential for ensuring optimal patient outcomes [39].

Factor 7, titled “Prevention and Promotion”, encompasses five key elements: 1. The doctor conducts a comprehensive assessment beyond my specific complaints. 2. I believe the doctor evaluates my risk of developing high-prevalence diseases. 3. The doctor suggests the most recommended preventive care for me, such as smoking cessation, weight loss, vaccinations, etc. 4. The doctor has a high degree of certainty that the suggested interventions will result in more benefits than harm. 5. The doctor initiates the most appropriate interventions to enhance my health. This factor underscores the importance of proactive measures aimed at reducing the risk of other diseases and promoting overall health and well-being. This includes encouraging positive behaviors, upholding honesty, considering social justice, utilizing resources wisely, and promoting the health of both patients and the public, all of which are fundamental standards of good medical practice [40].

Factor 8, titled “Patient’s Wants, Needs, and Satisfaction”, includes three primary elements: 1. The doctor understands my wants and addresses them. 2. The doctor recognizes my needs and fulfills them. 3. The doctor ensures my satisfaction with the care provided. This factor highlights the significance of patient satisfaction as a key indicator of the quality of health services [41].

The findings from the CFA significantly affirm the validity of the PEDMS’s structure, demonstrating excellent model fit as indicated by multiple fit indices: CFI (0.965), NFI (0.975), SRMR (0.051), and RMSEA (0.067). Additionally, the PEDMS exhibits strong convergent validity, supported by high CR and AVE values, both exceeding 0.70 and 0.50, respectively. Furthermore, all item loadings are greater than 0.70, reinforcing the PEDMS’s robustness. The reliability of the PEDMS is considered excellent, as demonstrated by a Cronbach’s alpha coefficient of 0.878 for the entire 38-item scale. Each factor exhibits strong internal consistency, with values ranging from 0.827 to 0.879, all surpassing the threshold of 0.7. This indicates that the items within each factor correlate well with one another, contributing to a cohesive and reliable measure. The “Cronbach’s Alpha if Item Deleted” values indicate that no individual item would substantially improve overall reliability; this information reinforces the internal consistency of the scale. The high reliability suggests that the scale consistently captures the underlying constructs it aims to assess, making it a trustworthy instrument for evaluating patient experiences with disease management. Such robust reliability is crucial for ensuring the validity of the findings derived from the scale, thereby enhancing its applicability in both clinical and research settings.

Moreover, several considerations highlight the uniqueness of the PEDMS. First, a comprehensive tool measures various aspects of health—physically, psychologically, and socially—in depth. This is achieved through organized domains of the management plan, each with logical consequences, ensuring a holistic approach to patient care. Second, while there are many reliable instruments, such as the Picker Patient Experience Questionnaire (PPE-15), Patient Experience Questionnaire (PEQ), National Health Service Inpatient Survey (NHSIP), and Hospital Consumer Assessment of Healthcare Providers and Systems (HCAHPS), that measure patient experiences primarily in inpatient and hospital settings [42], the PEDMS specifically targets outpatients—who represent the majority of healthcare service users. To the best of our knowledge, there is currently no standardized scale with satisfactory psychometric characteristics designed for this population. Third, the PEDMS is a patient-centered instrument that focuses primarily on patient-oriented outcomes rather than solely on health outcomes or health system metrics. This emphasis on the patient’s perspective ensures that care is tailored to individual needs and experiences, ultimately fostering better engagement and satisfaction in the management of their health. Such an approach is essential for enhancing the overall quality of care and improving patient experiences in healthcare settings. Fourth, the PEDMS employs a rigorous methodology to ensure its validity and reliability by integrating insights from the literature and conducting focus groups to explore personal perceptions in depth [43]. Additionally, face-to-face interviews were utilized during the psychometric phase to further validate the scale [44]. This comprehensive approach effectively identifies key thematic domains, ensuring that the item pool accurately reflects real-world experiences in disease management. Moreover, experts independently and systematically evaluated the items for clarity and relevance, ensuring content validity, while patient evaluations assessed face validity. This thorough process guarantees that the PEDMS is comprehensive, clear, and aligned with its intended purpose of assessment. Consequently, this methodology enhances the scale’s quality and validates its effectiveness for practical application in healthcare settings [45,46]. Furthermore, the PEDMS recognizes that quantitative data are essential for accurately assessing the constructs of interest. Interviewer-administered interviews ensured consistent data collection and improved the quality of the information gathered, making this approach particularly useful for initial assessments [19]. Fifth, responses are measured using a 5-point Likert scale, ranging from “strongly disagree” to “strongly agree”, facilitating a nuanced understanding of patients’ experiences and enabling effective analysis of response variations [47]. Last but not least, the evaluation of the PEDMS’s psychometric properties involved a large and diverse sample recruited through a probability sampling method, reflecting a wide range of demographics and medical conditions. The sample was evenly divided into two groups—one for EFA and the other for CFA. This design minimized measurement errors and achieved stable factor loadings, thereby enhancing the generalizability of the findings [48,49]. By identifying eight distinct factors that encompass the multifaceted nature of patient experiences, the PEDMS not only underscores the importance of comprehensive metrics in evaluating patient-centered care but also provides healthcare providers with actionable insights to foster quality improvement initiatives.

## 5. Conclusions

Collectively, these findings affirm that the developed scale is a well-structured instrument that effectively captures various dimensions of patient experience related to management plans. Its excellent model fit underscores its reliability and validity as a tool for assessing patient experiences in clinical practice. This scale provides valuable insights that can inform improvements in disease management strategies. Future research should focus on further validating the PEDMS across diverse outpatient populations and exploring its application in various healthcare contexts.

Proposed integration plan for PEDMS as a standardized tool:

The use of standardized measurements in healthcare, particularly in outpatient settings, is critical for providing consistent, high-quality patient care. Our proposed integration makes use of the PEDMS as a standardized tool for assessing patient experiences across diverse outpatient services. By implementing the PEDMS in these specific situations, healthcare providers can systematically gather and analyze patient feedback, identify areas for improvement, and make data-driven decisions to enhance patient-centered care. This integration is intended to address the following specific scenarios and cases:

1. Routine Check-Ups: Standardized metrics will help capture patient feedback on routine visits, ensuring that aspects such as appointment scheduling, waiting times, and overall satisfaction are consistently monitored and improved.
2. Chronic Disease Management: For patients managing chronic conditions, the PEDMS will provide insights into their ongoing care experiences, including the effectiveness of communication with healthcare providers, the coordination of care, and the support received for self-management.
3. Specialized Treatments: In cases where patients receive specialized treatments (e.g., oncology and cardiology), the PEDMS will help assess the quality of care, patient-provider interactions, and the adequacy of information provided about treatment options and outcomes.
4. Post-Treatment Follow-Ups: The scale will be used to evaluate patient experiences during follow-up visits, focusing on the continuity of care, the clarity of post-treatment instructions, and the overall support provided during recovery.

## 6. Strengths and Limitations

The article presents several strengths, including a comprehensive methodology that integrates qualitative and quantitative approaches, a thorough literature review supporting the identification of key dimensions of patient experience, and rigorous psychometric testing through exploratory and confirmatory factor analyses, which enhance the reliability and validity of the PEDMS. It also benefits from a diverse sample of 600 outpatients with various demographic features and health conditions. However, limitations exist, such as this study being conducted in a single setting, which may restrict generalizability. Additionally, the absence of criterion validity testing limits insights into the scale’s predictive capabilities, while the cross-sectional design restricts the assessment of changes in patient experiences over time. To measure patient experiences with disease management, it is vital to classify diseases, for example, healthcare-associated infections (HAIs) and others; however, the classification could not be included. Lastly, translation issues may affect item interpretation across diverse populations and cultures, indicating areas for future research. Future research could explore the integration of digital healthcare components into the PEDMS to provide a more comprehensive assessment of patient experiences in both traditional and digital outpatient settings.

## Figures and Tables

**Table 1:** Characteristics for all study’ samples (*n* = 600).

		*n*	%
Gender	Male	243	40.5
	Female	357	59.5
Age Mean (±SD) 33.74 (±14.69)	20 years or less	121	20.2
	21–40 years	281	46.8
	Above 40 years	198	33.0
Marital status	Single	202	33.7
	Married	386	64.3
	Divorced	8	1.3
	Widow	4	0.7
Nationality	Saudi	336	56.0
	Non-Saudi	264	44.0
Education	Illiterate	29	4.8
	General education—primary/intermediate/secondary	263	43.8
	University education—diploma/bachelor	292	48.7
	Postgraduate education—master’s/Ph.D.	6	1.0
	Other	10	1.7
Residency	Rural	66	11.0
	Semiurban	289	48.2
	Urban	245	40.8
Family type	Nuclear	88	14.7
	Extended	512	85.3
Family member number	<3	53	8.8
	From 3 to 7	445	74.2
	From 8 to 14	91	15.2
	>15	11	1.8
Employment	Nothing	314	52.3
	Governmental	138	23.0
	Private	136	22.7
	Nonprofit	1	0.2
	Self-employed	11	1.8
Economic status	Extremely low	30	5.0
	Low	176	29.3
	Moderate	338	56.3
	Above moderate	56	9.3

**Table 2:** Domain identification and conceptual descriptions.

Domain	Description
Reassurance/explanation	Providing patients with clear, concise information about their health conditions and treatment plans, along with realistic hope and emotional support.
Advice/counseling	Tailoring recommendations based on individual patient needs, preferences, and circumstances enhances overall well-being and empowers patients to take an active role in their care.
Prescribing	Selecting appropriate medications, determining dosages, providing clear instructions for use, discussing potential side effects, and emphasizing the importance of adherence.
Red flags/referral	A proactive approach ensures that patients can identify warning manifestations that indicate potentially serious underlying conditions requiring further evaluation and that they understand the rationale for referral.
Investigation	Ensuring that patients understand the purpose and significance of the tests, imaging, and procedures being conducted.
Observation/follow-up	An ongoing process of monitoring a patient’s condition involves detecting any changes in health status and making necessary adjustments to the treatment plan.
Prevention/promotion	Proactive measures aimed at reducing the risk of other diseases and enhancing overall health and well-being include encouraging positive behaviors.
Patient’s wants/needs/satisfaction	Understanding and addressing individual preferences and requirements in the healthcare experience, along with considering patient satisfaction.

**Table 3:** Factor loading results from exploratory factor analysis (EFA) (*n* = 300).

Items	Factor Loading								Cronbach’s Alpha	Cronbach’s Alpha If Item Deleted
	1	2	3	4	5	6	7	8		
**Reassurance and Explanation**										
1. The doctor treats my need for reassurance as the primary reason for seeking medical services.	0.788								0.876	0.872
2. I receive appropriate reassurance that maintains the doctor’s credibility.	0.789									0.871
3. The doctor provides effective reassurance through thorough history taking and examination.	0.907									0.874
4. The doctor offers an appropriate degree of explanation regarding my illness.	0.918									0.872
5. The doctor explores my understanding and fears concerning the symptoms.	0.919									0.873
6. I trust the doctor due to effective communication.	0.942									0.874
7. The doctor considers my understanding, education, cultural background, medical experience, and personality.	0.873									0.872
8. I have a strong bond with the doctor based on continuity of care.	0.927									0.874
**Advice and Counseling**										
1. The doctor’s advice is realistically adapted to my circumstances, lifestyle, and personality.				0.766					0.835	0.828
2. The doctor helps me identify the physical, psychological, and social aspects of my illness.				0.767						0.821
3. The doctor assists me in implementing my own solutions for my condition by providing insights and identifying possible actions.				0.729						0.819
4. The doctor convinces me that I am not physically or psychologically ill; rather, I am facing challenges in adapting to or coping with everyday problems.				0.631						0.815
5. The doctor counsels me to recognize the need to modify unhealthy behaviors.				0.633						0.821
**Prescribing**										
1. I believe the doctor considers any warnings regarding medication safety before prescribing.					0.665				0.840	0.827
2. I think the doctor prescribes the most effective medication.					0.599					0.828
3. I believe the doctor takes into account the availability and affordability of medication before prescribing.					0.740					0.827
4. The doctor informs me about the possible adverse effects of medication prior to prescribing.					0.660					0.824
5. The doctor instructs me on the dosage, timing, and route of administration for the medication.					0.730					0.827
**Red Flags and Referral**										
1. The doctor makes me aware of the signs that may indicate serious progression or complications that could occur.			0.789						0.851	0.839
2. The doctor explains how I can manage if I encounter any potential serious progression or complications.			0.876							0.835
3. I believe the doctor arranges appropriate referrals when necessary.			0.903							0.840
4. The doctor details the referral process, including why, to whom, when, and where the referral will take place.			0.765							0.840
5. The doctor seeks feedback regarding the referral.			0.773							0.839
**Investigation**										
1. I believe the doctor requests appropriate investigations							0.757		0.836	0.821
2. I think the doctor considers the risks and costs justified by the value of the information likely to be gained from the tests.							0.707			0.813
3. I understand the purpose of the requested investigations.							0.595			0.825
4. The doctor discusses with me what is being looked for in the results.							0.757			0.809
**Observation and Follow-up**										
1. The doctor encourages follow-up visits to monitor progress.						0.774			0.879	0.873
2. I believe the doctor schedules appropriate appointments for follow-up.						0.774				0.871
3. The doctor effectively addresses any issues related to follow-up.						0.779				0.864
**Prevention and Promotion**										
1. The doctor conducts a comprehensive assessment beyond my specific complaints.		0.938							0.840	0.821
2. I believe the doctor evaluates my risk of developing high-prevalence diseases.		0.863								0.818
3. The doctor suggests the most recommended preventive care for me, such as smoking cessation, weight loss, vaccinations, etc.		0.620								0.836
4. The doctor has a high degree of certainty that the suggested interventions will result in more benefits than harm.		0.725								0.826
5. The doctor initiates the most appropriate interventions to enhance my health.		0.534								0.831
**Patient’s wants, needs, and satisfaction**										
1. The doctor knows my wants and treat them.								0.639	0.827	0.821
2. The doctor knows my need and replenishes it.								0.839		0.812
3. The doctor accomplishes my satisfaction.								0.715		0.801

Extraction method: principal component analysis. Rotation method: Promax with Kaiser normalization ^a^. ^a^ Rotation converged in 8 iterations.

**Table 4:** Model fit measures for CFA model (*n* = 300).

Criteria	CFA Model	Thresholds
CMIN	1015.378	--
DF	637	--
CMIN/DF	1.594	Between 1 and 3
*p*-value	***	Between 0.05 and 0.000
CFI	0.965	>0.95
NFI	0.975	>0.95
TLI	0.973	>0.95
GFI	0.952	>0.90
AGFI	0.938	>0.90
SRMR	0.051	<0.08
RMSEA	0.067	<0.08

Note: The criterion thresholds and their interpretations were based on Hu and Bentler (1999) [24]. *** *p* < 0.001, The *** symbol indicates that the p-value is highly significant (*p* < 0.001).

**Table 5:** Average variance extracted (AVE).

	Factor 1	Factor 2	Factor 3	Factor 4	Factor 5	Factor 6	Factor 7	Factor 8
Factor 1	0.859							
Factor 2	0.767 ***	0.869						
Factor 3	0.524 ***	0.551 ***	0.795					
Factor 4	0.669 ***	0.671 ***	0.518 ***	0.883				
Factor 5	0.828 ***	0.733 ***	0.542 ***	0.758 ***	0.773			
Factor 6	0.651 ***	0.608 ***	0.615 ***	0.623 ***	0.620 ***	0.848		
Factor 7	0.704 ***	0.608 ***	0.622 ***	0.700 ***	0.754 ***	0.612 ***	0.886	
Factor 8	0.768 ***	0.683 ***	0.531 ***	0.677 ***	0.748 ***	0.526 ***	0.617 ***	0.878
CR	0.894	0.903	0.837	0.914	0.815	0.885	0.936	0.910
AVE	0.738	0.756	0.631	0.779	0.598	0.719	0.786	0.771

Note: CR = construct reliability; AVE = average variance extracted. Measures on the diagonal are square roots of AVEs, and measures below the diagonal are correlation values among the constructs. *** = *p* < 0.001.

## Data Availability

The data presented in this study are available on request from the corresponding author.
